# From Dual Decline to Clinical Consequences: Mechanistic Pathways and Health Outcomes of Motoric Cognitive Risk Syndrome

**DOI:** 10.14336/AD.2025.0730

**Published:** 2026-09-03

**Authors:** Yiwei Zhao, Tao Wei, Bo Zhao, Yuanyuan Chen, Zhibin Wang, Yi Tang

**Affiliations:** ^1^Department of Neurology and Innovation Center for Neurological Disorders, Xuanwu Hospital, National Center for Neurological Disorders, Capital Medical University, 45 Changchun Street, Beijing, 100053, China; ^2^Neurodegenerative Laboratory of Neurodegenerative Laboratory of Ministry of Education of the People’s Republic of China, Beijing, 100053, China

**Keywords:** Motoric Cognitive Risk Syndrome, Dementia, Frailty, Falls, Mortality, Interventions

## Abstract

Motoric cognitive risk (MCR) syndrome, defined by the coexistence of slow gait and subjective cognitive decline (SCD), is increasingly recognized as an intermediate stage between normal aging and overt cognitive impairment. Its prevalence varies across populations, largely shaped by demographic, socioeconomic, and lifestyle factors. Neuroimaging evidence further supports this construct, demonstrating gray matter atrophy in prefrontal and premotor regions alongside white matter hyperintensities, both of which have been linked to motor and cognitive deficits in individuals with MCR. The pathogenesis of MCR is likely multifactorial, involving metabolic, inflammatory, and genetic mechanisms that interact to accelerate vulnerability to neurodegeneration. Importantly, MCR has been shown to predict adverse outcomes, including dementia, falls, frailty, and increased mortality, underscoring its potential utility as an early clinical marker of neurodegenerative progression. Given these implications, recent studies have explored preventive and therapeutic strategies, such as aerobic exercise, dual-task cognitive training, dietary interventions, and pharmacological approaches targeting inflammation and metabolic dysfunction, that may mitigate MCR-related risks. Future research should aim to refine diagnostic criteria, integrate multimodal assessments, and develop personalized interventions to more effectively prevent or delay cognitive and motor decline in aging populations.

## 1. Introduction

Dementia prevalence is rising worldwide. The Global Burden of Disease (GBD) 2019 forecasting study, published in The Lancet Public Health in 2022, estimated that the number of affected individuals will reach about 152 million by 2050 [1]. More recent analyses based on the GBD database up to 2021 have reinforced and refined these projections, confirming the continuing upward trend in dementia burden [2]. Dementia reduces both quality of life and life expectancy. Early detection strategies currently include biomarker testing, neuroimaging, and specialized cognitive assessments. Recently, the coexistence of slow gait and subjective cognitive decline (SCD) has been recognized as a potential early marker of dementia [3], providing opportunities for earlier intervention. Motoric Cognitive Risk (MCR) syndrome is defined by the coexistence of slow gait and SCD in older adults who remain cognitively intact and functionally independent [3]. Evidence from multiple international studies indicates that MCR is associated with a markedly increased risk of dementia and other adverse aging outcomes [4].

Despite its growing recognition, important gaps remain in our understanding of the neurobiological mechanisms underlying MCR and the effectiveness of potential interventions. The objectives of this review are to: (1) evaluate current methods for assessing MCR, (2) examine its neurobiological basis, (3) identify modifiable risk factors, and (4) assess its predictive value for adverse outcomes, based on evidence from a structured literature search. We also propose targeted intervention strategies to improve early recognition and emphasize the need for multidisciplinary collaboration. These efforts aim to inform prevention approaches and support public health policies addressing dementia and related geriatric conditions.

## 2. Methods

We conducted a literature search in PubMed, Web of Science, Embase, and the VIP Journal Database, covering all records from database inception to January 1, 2025, to provide a comprehensive overview of MCR.

The search strategy combined Medical Subject Headings (MeSH) and free-text keywords with Boolean operators (OR, AND). Representative terms included: "old*" OR "elder*" OR "senior*", "prevalence*" OR "incidence*" OR "epidemiology*" OR "number*" OR "risk*", "walking speed*" OR "slow gait*" OR "physical function*" OR "gait disorder*" OR "motor dysfunction*" OR "motor function*" OR "gait speed*", "subjective cognitive concern*" OR "subjective cognitive decline*" OR "cognitive complaint*", and "MCR" OR "motoric cognitive risk*" OR "motoric cognitive risk syndrome*".

The strategy was first developed in PubMed and then adapted for the other databases. Reference lists of relevant articles and reviews were also screened manually. All records were imported into EndNote for deduplication. Two reviewers independently screened titles and abstracts, with full texts retrieved for potentially eligible studies. Disagreements were resolved by discussion or, if required, a third reviewer. Inter-reviewer agreement was assessed using Cohen’s kappa.

### 2.1 Study Selection

Studies were selected based on relevance to the objectives of this review. We prioritized original research—cohort, cross-sectional, and longitudinal studies—that examined MCR as an exposure and reported outcomes such as dementia, cognitive impairment, falls, mortality, disability, or frailty. A “comprehensive evaluation of MCR characteristics” was defined as the simultaneous assessment of both diagnostic components: slow gait (gait speed <1.0 m/s or below age- and sex-specific norms) and subjective cognitive complaints (assessed by validated self-report or informant-based tools). Eligible studies had to report at least one clinical or functional outcome, including dementia, cognitive decline, falls, disability, frailty, or mortality. To provide broader context, relevant reviews and book chapters were also considered.

Case reports, ecological studies, and experimental studies were excluded if their designs did not allow a comprehensive evaluation of MCR or related outcomes. Brief reports, editorials, and they were also excluded, unless they provided unique and highly relevant insights.

## 3. Epidemiology and Concept of MCR

Aging is a multifaceted biological process that affects cognitive function, motor performance, and multiple organ systems. Motoric Cognitive Risk (MCR) has been recognized as a critical transitional stage between normal aging and cognitive impairment, characterized by the concurrent decline in both cognitive and motor domains. Its diagnostic criteria—slow gait and subjective cognitive decline (SCD)—not only capture underlying neurodegenerative processes but also independently predict incident dementia [5]. From a gerontological perspective, identifying MCR allows the early recognition of high-risk individuals and underscores the need for integrated motor-cognitive interventions to promote healthy aging [6, 7].

Epidemiological research has demonstrated considerable global variability in MCR prevalence. Systematic analyses estimate an overall prevalence of approximately 9.70% (95% confidence interval [CI] = 8.20%-11.20%) [6], with marked differences across geographic regions and population subgroups. Such variation likely reflects both the application of heterogeneous diagnostic criteria and differences in study populations.

Prevalence rates vary markedly by region [8]. In Asia, reported prevalence ranges from 7.46% to 13.60% [9, 10], while European studies report lower rates, between 2.56% and 9.92% [11, 12]. North American populations show the broadest range, with estimates spanning from 4.22% to 14.74% [13]. Low-income countries and regions generally report higher prevalence, ranging from 5.30% to 15.60% [14, 15]. In addition to geographic and socioeconomic influences, certain high-risk groups show elevated MCR prevalence. These include individuals with lower educational attainment [16], those leading sedentary lifestyles or engaging in low levels of physical activity [17-19], and individuals with cardiovascular diseases or chronic inflammation [20, 21].

Beyond geographic and population factors, differences in MCR prevalence are largely attributable to inconsistencies in assessment methods. Gait speed measurement is a central component, and variations in testing distance (2.5 m, 4-6 m), devices (GAITRite vs. stopwatch), start-stop procedures, and allowance of walking aids can all introduce systematic bias. For example, the CHARLS study (China), which employed a 2.5 m short-distance test, reported an MCR prevalence of approximately 9-13%, markedly higher than the 2-5% observed in the TILDA study (Ireland, GAITRite 4.88 m) [11, 14]. The operational definition of “slow gait” also directly influences prevalence estimates: in the NuAge cohort (Canada), using an age- and sex-specific 1 SD threshold yielded a prevalence of only about 4-7%, whereas studies applying a fixed cut-off of <0.8 m/s frequently reported rates of 10-15% [12, 13]. Differences in measuring subjective cognitive complaints further contribute to variability, with single-item measures often underestimating prevalence, while multi-item scales or objective tasks significantly increased case detection [13, 17]. In addition, whether participants using walking aids were excluded, and the definitions applied for dementia or disability, also alter sample composition and overall estimates [13, 21]. Therefore, methodological heterogeneity in assessment protocols and diagnostic thresholds is not a mere technical detail but a critical determinant of the reported prevalence of MCR across epidemiological studies and regions.

## 4. Structural Brain Changes and Pathological Mechanisms of MCR

The association between MCR and adverse outcomes such as cognitive decline, falls, and mortality appears to result from the interaction of multiple biological pathways rather than a single mechanism. These mechanisms may act synergistically, and a bidirectional relationship may exist between the development of MCR and subsequent adverse outcomes. Although no single pathway fully explains MCR pathophysiology, current evidence highlights several converging biological processes [22].

### 4.1 Neuroanatomical alterations

Recent studies have advanced understanding of the neurobiological basis of MCR. Neuroimaging investigations have consistently demonstrated characteristic structural brain alterations, most notably reduced gray matter volume in the prefrontal and premotor cortices, including the dorsolateral prefrontal cortex (DLPFC) [22]. Atrophy of the DLPFC is particularly noteworthy given its central role in executive function, memory processing, and gait regulation [23]. These changes have been proposed as neuropathological substrates of MCR [24] and may account for the combined impairments in executive function and gait observed in affected individuals [25-27]. Such neuroanatomical abnormalities correlate with the core clinical features of MCR, suggesting disruption of prefrontal-motor cortical networks [28]. This supports the hypothesis that MCR may represent a prodromal stage of dementia, especially frontal-variant Alzheimer’s disease (AD) or Lewy body dementia, both of which involve early cortical dysfunction. In addition, individuals with MCR frequently exhibit cerebral small vessel disease, including white matter hyperintensities, lacunar infarcts, and cerebral microbleeds [29]. These vascular lesions may accelerate cognitive-motor decline by reducing cerebral perfusion and disrupting large-scale neural networks [30].

### 4.2 Cholinergic dysfunction

Direct investigations of the cholinergic system in MCR populations are lacking; however, research in neurodegenerative disorders such as AD and Parkinson’s disease (PD) provides relevant insights [31, 32]. In AD, degeneration of basal forebrain cholinergic neurons is a hallmark pathological feature [33]. Downregulation of cholinergic markers has also been observed in asymptomatic older adults, suggesting that cholinergic dysfunction may occur early in the neurodegenerative cascade [34]. Data from the Alzheimer’s Disease Neuroimaging Initiative (ADNI) further support this view: cholinergic atrophy correlates with disease progression, with patients diagnosed with late mild cognitive impairment (LMCI) showing greater cholinergic volume loss than those with early mild cognitive impairment (EMCI) [35]. Although direct evidence in MCR is not yet available, the syndrome is characterized by progressive cognitive-motor decline and shares clinical features with both AD and PD. These parallels imply that cholinergic dysfunction could contribute to MCR, a hypothesis requiring direct validation.

### 4.3 Genetic and inflammatory mechanisms

Genetic and inflammatory factors also appear to influence MCR susceptibility. A large-scale European study on the polygenic architecture of MCR found that genetic profiles associated with obesity are linked to increased risk of developing the syndrome [36]. Other studies have identified associations between inflammatory cytokine polymorphisms and MCR. In particular, IL-10 polymorphisms have been implicated: experimental evidence suggests that IL-10 overexpression may promote amyloid-beta (Aβ) deposition, impair microglial clearance, and alter APOE expression, thereby affecting cognitive-motor outcomes [37, 38]. The *APOE* ε4 allele, a well-established genetic risk factor for MCR, has been independently linked to slower gait speed and greater disability risk in older adults [39, 40]. Notably, unlike in mild cognitive impairment (MCI), IL-10 polymorphisms in MCR appear more strongly linked to motor rather than purely cognitive pathways, which may help explain its distinct phenotype [38].

### 4.4 Metabolic and vascular factors

Chronic diseases and metabolic dysregulation represent additional systemic contributors to MCR [41]. Hypertension increases arterial stiffness, leading to cerebral hypoperfusion and compromising white matter integrity [42]. Type 2 diabetes mellitus (T2DM) further exacerbates cerebrovascular injury through mechanisms such as glycemic variability and oxidative stress [42]. Features of metabolic syndrome—including insulin resistance and mitochondrial dysfunction—disrupt connectivity within the default mode network, particularly in the prefrontal and posterior cingulate cortices, thereby impairing cognitive-motor performance. Obesity may also promote MCR by altering the functional organization of large-scale neural networks.

Overall, the pathogenesis of MCR is multifactorial and multilayered, encompassing neurostructural changes, neurotransmitter alterations, genetic and inflammatory factors, and systemic metabolic disturbances. These findings underscore the need for interventions targeting neuroprotection, vascular health, metabolic regulation, and inflammation. Future research should clarify how these mechanisms interact, thereby establishing a stronger foundation for precise diagnosis and targeted treatment of MCR.

**Table 1:** Risk Factors of MCR.

Risk Factor Category	Specifics	Mechanisms
Lifestyle Factors	Physical Exercise	Lack of physical activity reduces cerebral blood flow perfusion and neurotrophic factor secretion, while increasing inflammatory responses. This impairs cognitive and motor functions. Sedentary behavior and decreased muscle strength further reduce gait stability and hinder metabolic waste clearance, accelerating progression of MCR [43].
	Diets	Dietary patterns exert protective effects on cognitive function through antioxidants (e.g., omega-3 fatty acids) and anti-inflammatory actions while improving cerebral microcirculation. Deficiencies in key nutrients (B vitamins, vitamin C, etc.) also impair cerebral metabolic function and accelerate cognitive-motor decline [44].
Multimorbidity	Cardiovascular and Metabolic Conditions	Cardiometabolic diseases impair cerebrovascular function (e.g., endothelial dysfunction, impaired cerebral microcirculation) and induce chronic inflammatory states, which contributes to MCR development [45].
	Pulmonary Functions	Impaired pulmonary function contributes to cognitive-motor decline via chronic hypoxia, systemic inflammation, and reduced physical activity [46].
	Sleep Disorders	Sleep disorders increase risk of MCR by damaging memory-related neural circuits, reducing Aβ clearance efficiency, and disrupting the dopamine system [47].
	Sensory Impairments	Visual/hearing impairments increase MCR risk via attentional load, sensory deprivation-induced cortical reorganization, and shared neurodegeneration [48].
	Chronic Pain	Pain contributes to MCR development via two primary pathways: competition for attentional resources and induction of prefrontal cortex remodeling. Neuroimaging evidence shows prefrontal atrophy in MCR [49].
	Polypharmacy	Polypharmacy elevates MCR risk by impairing both cognitive functions and motor coordination through individual drug effects or combined interactions [41].
Social/Environmental Factors	Social Supports	Social engagement protects against MCR by promoting synaptic connectivity, supporting hippocampal-prefrontal network integrity, and reducing neural network decline [50].
	Environmental and Psychological Stress Effects	Environmental stressors (e.g., noise pollution) are significantly associated with hippocampal atrophy and slow gait. Perceived stress and adverse childhood experiences also promote the development of MCR through hypothalamic-pituitary-adrenal (HPA) axis dysregulation [51].

Abbreviations: MCR, Motoric Cognitive Risk Syndrome.

## 5. Risk Factors of MCR

**Figure 1.**
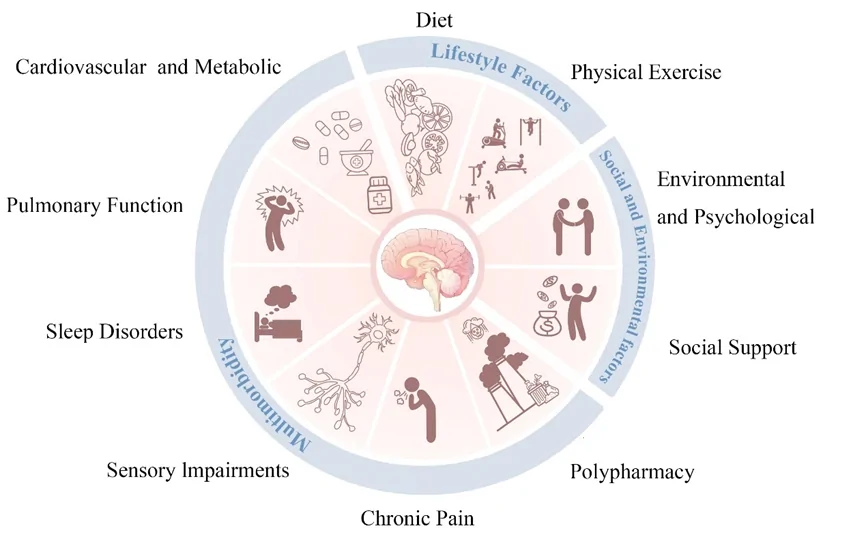
**Risk factors of MCR**. Both known and potential risk factors of MCR are highlighted, including lifestyle factors, multimorbidity, and social and environmental factors. Abbreviations: MCR, Motoric Cognitive Risk Syndrome.

The development of MCR reflects interactions among lifestyle, metabolic, and environmental factors. Figure 1 summarizes potential contributors. Regular physical activity and adherence to a Mediterranean diet may lower risk by improving cerebral perfusion and reducing neuroinflammation. In contrast, comorbidities such as type 2 diabetes mellitus (T2DM) and sensory impairments markedly increase susceptibility. Reduced social engagement and chronic environmental stressors, including noise exposure, have also been linked to MCR in epidemiological studies. These modifiable factors likely promote MCR by impairing both brain structure and functional connectivity, driving parallel declines in cognition and motor function. Table 1 provides a summary of key risk factors and underlying mechanisms.

### 5.1 Lifestyle Factors: Physical exercise and diet

#### 5.1.1 Physical Exercise

Physical activity is essential for maintaining cognitive and motor health. Aerobic exercises such as walking and swimming enhance cerebral blood flow and support neuroplasticity, while resistance training improves gait stability, strengthens muscles, and lowers fall risk [52]. In individuals with MCR, regular exercise helps preserve cognition by improving cerebral hemodynamics. Functional near-infrared spectroscopy (fNIRS) studies have shown intensity-dependent increases in oxygenated hemoglobin (OxyHb) within the prefrontal cortex during submaximal cycling, with levels correlating with perceived exertion [53]. These findings provide direct evidence that exercise promotes cerebrovascular adaptation, supporting its neuroprotective role [54].

Sedentary behavior, in contrast, is consistently associated with higher MCR risk [55]. The benefits of physical activity are mediated through several pathways [56, 57]. Exercise lowers cardiovascular risks such as hypertension, insulin resistance, and hyper-cholesterolemia, while also enhancing immune function, reducing inflammation, and increasing secretion of neurotrophic factors [58].

#### 5.1.2 Diet

Dietary patterns and nutrient intake influence both cognitive and motor decline in MCR [59]. The Mediterranean diet and the Dietary Approaches to Stop Hypertension (DASH) diet are considered protective due to their high content of antioxidants [60], polyphenols, and healthy fats. Specific components of the Mediterranean diet—such as olive oil polyphenols, berry-derived antioxidants, and marine omega-3 fatty acids—may reduce oxidative stress and inflammation [61], enhance cerebral microvascular circulation, and slow neurodegenerative processes [62]. The DASH diet may also lower risk indirectly by reducing blood pressure and improving cerebral blood flow [63].

Nutritional risk is strongly associated with cognitive-motor decline in MCR. Patients at higher risk of malnutrition perform worse in working memory, executive function, and global cognition than those with adequate nutrition [64]. In older populations, insufficient caloric intake (<1500 kcal/day) and low dietary fiber may accelerate both cognitive decline and gait disturbances [65]. A cross-sectional analysis of older adults with MCR reported that 36% of participants were at risk of malnutrition, defined by a Mini Nutritional Assessment score below 24 [66]. Nutritional risk scores correlated negatively with working memory, executive function, and global cognition [67]. Although dietary quality was moderate according to U.S. guidelines, only 20% adhered closely to the Mediterranean-DASH Intervention for Neurodegenerative Delay (MIND) diet, while 48% showed poor adherence. Intake of B vitamins (e.g., B6 and folate) and vitamin C correlated positively with task-switching speed and processing efficiency, suggesting that specific micronutrients may slow cognitive decline by supporting brain metabolism and antioxidant defenses [68, 69].

### 5.2 Multimorbidity

The development and progression of MCR are shaped by multisystem interactions. Recent large cohort studies have clarified a range of risk factors and mechanisms associated with this syndrome.

Cardiometabolic multimorbidity is a major contributor. Studies have shown strong associations between the coexistence of multiple metabolic conditions and MCR risk [70-72]. Data from the China Health and Retirement Longitudinal Study (CHARLS) revealed that the coexistence of hypertension, diabetes, and coronary heart disease elevated MCR risk by 57% [73]. Individuals with multimorbidity had a 41% higher risk (HR = 1.41, 95% CI = 1.13-1.75), and a dose-response pattern was evident: each additional cardiometabolic condition raised risk by 33% (HR = 1.33, 95% CI = 1.20-1.48) [45].

Pulmonary function also plays an important role. Peak expiratory flow (PEF) has been identified as an independent protective factor against MCR. Cross-sectional studies found lower risk among individuals in higher PEF tertiles (highest tertile OR = 0.66, 95% CI = 0.52-0.84). Percentile-based analysis showed that those with PEF standard residuals (SR) below the 30th percentile had a 3.04-fold higher risk compared with the reference group (95% CI = 1.85-5.01) [74]. Longitudinal follow-up confirmed these associations: the highest tertile had a 42.40% lower risk (HR = 0.57, 95% CI = 0.43-0.76) and delayed onset by 0.484 years (95% CI = 0.15-0.81), whereas the lowest PEF SR group showed an 81% higher six-year cumulative risk (HR = 1.81, 95% CI = 1.24-2.66). These findings provide clinical support for the “lung-brain axis” hypothesis [74].

Sensory system dysfunction is another important factor. Visual impairment, hearing impairment, and dual sensory impairment have each been linked to higher MCR risk [48]. This may be due to reduced cognitive stimulation and impaired sensory-motor integration. Hearing impairment is recognized as a modifiable risk factor, with correction estimated to prevent around 8% of dementia cases [48]. Proposed mechanisms include perceptual cognitive load, sensory deprivation, and the common cause theory. Intervention studies show that sensory improvements, such as hearing aids, can delay cognitive decline.

Sleep disorders are also associated with MCR. Daytime sleepiness has been identified as an independent risk factor [75]. In older Chinese populations, moderate napping (30-89 minutes) is common and linked to lower MCR risk. Several studies report a U-shaped relationship between nap duration and cognitive performance, with 30-60 minutes associated with the greatest benefit, while both shorter and longer naps predict adverse outcomes [76]. Mechanistic explanations include disrupted hippocampal-prefrontal plasticity, reduced cerebrospinal fluid clearance of Aβ caused by sleep fragmentation, and circadian rhythm disturbances affecting dopaminergic function [77].

Chronic pain and polypharmacy deserve particular attention. Cross-sectional data show that individuals experiencing interfering pain have a significantly higher risk of developing MCR (OR = 1.51, 95% CI = 1.17-1.95; p = 0.001). Longitudinal follow-up further supports this association, demonstrating that participants with baseline interfering pain faced an increased risk of MCR over an 8-year period (HR = 2.02, 95% CI = 1.52-2.69; p < 0.001) [49]. The interruptive pain model suggests that pain competes for attentional resources and impairs executive function [49]. Neuroimaging studies further show that chronic pain remodels the prefrontal cortex, a region also reduced in individuals with MCR [49, 78]. These observations suggest a bidirectional relationship, where pain accelerates cognitive-motor decline and declining cognitive resources heighten pain perception [79]. Recognizing this reciprocal relationship highlights the need for integrated interventions addressing both pain management and cognitive-motor health [80].

Polypharmacy is significantly associated with motoric MCR in older adults. Studies report a higher prevalence of MCR in individuals with polypharmacy (10%) compared to those without (6%), with affected individuals being 1.8 times more likely to meet MCR criteria (OR = 1.80, 95% CI = 1.0-3.0; *p* = 0.03). Associations are seen with both baseline MCR (OR = 1.27, 95% CI = 1.05-1.54; p < 0.05) and incident risk, which increases in a dose-dependent manner. Each additional medication raises risk by 53.80% (HR = 1.53, 95% CI = 1.22-1.92; p < 0.001) [81, 82]. Three interrelated mechanisms likely explain this relationship. First, specific drug classes such as anticholinergics and proton pump inhibitors may impair cognitive function through direct effects on the central nervous system. Second, side effects of medications may disrupt the integrated functioning of the respiratory, cardiovascular, and neuromuscular systems, thereby reducing gait speed. Third, drug-drug interactions may simultaneously impair cognitive and motor functions, contributing to the core clinical manifestations of MCR.

Overall, current evidence indicates that effective prevention and management of MCR syndrome require integrated strategies, including metabolic regulation, sensory function support, sleep quality improvement, and appropriate medication use. Future studies should examine the interactions among these risk factors to guide the development of precision-based prevention strategies [83].

### 5.3 Social and Environmental Factors

The neuroprotective effects of social engagement are well documented. Active participation appears to delay cognitive decline by promoting neural plasticity and preserving the integrity of neural networks. Studies consistently report an inverse association between strong social support and MCR incidence. In a group-based multi-trajectory modeling (GBMTM) analysis (n = 2,279), researchers identified four distinct levels of social support (low, medium, high, and high with employment), which corresponded with a graded reduction in MCR prevalence (9.4%, 9.0%, 4.1%, and 0.8%, respectively). After adjusting for multiple variables, individuals in the low and medium support groups had significantly higher MCR risks—4.07-fold (aOR = 4.07, 95% CI = 1.60-10.34) and 3.10-fold (aOR = 3.10, 95% CI = 1.26-7.66), respectively—compared to those in the high-employment group. These results establish a clear dose-response relationship between social support and MCR risk [84], providing critical evidence for designing targeted psychosocial interventions.

Psychosocial stressors are also emerging as contributors to MCR. Epidemiological data highlight perceived stress and adverse childhood experiences as risk factors, potentially acting through dysregulation of the hypothalamic-pituitary-adrenal (HPA) axis. In parallel, environmental exposures such as noise pollution have received increasing attention. Neuroimaging evidence shows negative associations between hippocampal volume and both MCR risk and gait speed decline among individuals with preserved high-frequency hearing [85]. These results suggest that environmental stressors can affect brain structure and function in ways that influence MCR development.

## 6. Diagnostic Criteria for MCR

**Figure 2.**
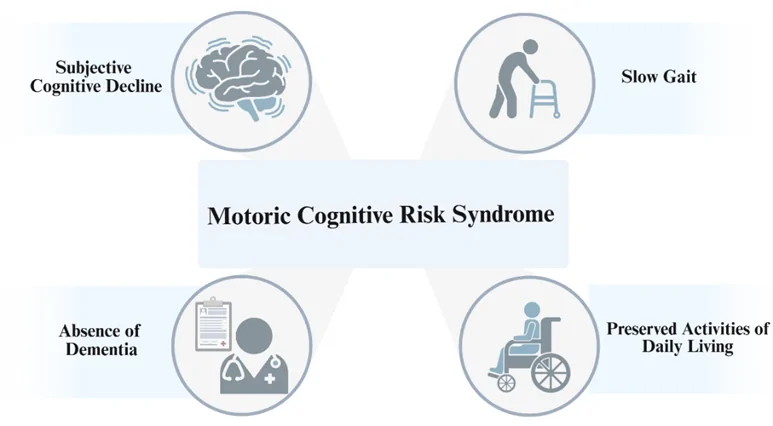
**Diagnostic Criteria for MCR**. Diagnosis requires both subjective cognitive decline and slow gait, in the absence of dementia and with preserved Activities of Daily Living (ADL). Each component is evaluated using standardized tools such as structured interviews, gait speed tests, and ADL scales. Abbreviations: MCR, Motoric Cognitive Risk Syndrome; ADL, Activities of Daily Living.

The diagnostic criteria for MCR syndrome include four essential components, all of which must be met: the presence of SCD, slow gait, absence of a dementia diagnosis, and preserved activities of daily living. The co-occurrence of cognitive and motor impairment defines the core clinical feature of MCR (Fig. 2).

Subjective Cognitive Decline (SCD): Self-reported concerns regarding memory or other cognitive functions, typically assessed using standardized questionnaires or structured clinician interviews.

Slow Gait: Gait speed measured as at least one standard deviation below the age- and sex-adjusted population norm, evaluated using instrumented gait analysis or timed walking tests.

Absence of Dementia: No clinical diagnosis of dementia based on criteria from the ICD-11/10 or the Diagnostic and Statistical Manual of Mental Disorders, 4th edition (DSM-IV).

Preserved Activities of Daily Living (ADL): Minimal or no impairment in daily functional abilities, confirmed using validated ADL assessment scales.

These criteria allow the identification of individuals at risk for MCR in both clinical and community settings. However, the diagnostic process faces several limitations, particularly regarding inter-study comparability and clinical utility. For example, assessments of SCD are susceptible to self-reporting bias. Incorporating standardized questionnaires can mitigate this limitation and improve the accuracy of early detection. Similarly, inconsistencies in gait assessment methods hinder reproducibility. To address this, we recommend adopting standardized quantitative protocols, such as electronic gait analysis in combination with the 4-meter walking test, to improve reliability and comparability.

### 6.1 Assessment Methods and Diagnostic Thresholds for Slow Gait

Gait speed assessment is a key measure of motor function in MCR and an established predictor of health outcomes in older adults. Often referred to as the “sixth vital sign” [86], gait speed closely correlates with life expectancy. A 2018 meta-analysis including more than 100,000 older adults with a median follow-up of 5.4 years reported that each 0.1 m/s decrease in gait speed was linked to a 12% higher risk of early mortality (HR = 1.12, 95% CI = 1.09-1.14) [87]. In MCR, gait speed is typically measured using either manual stopwatches or automated devices such as pressure-sensitive walkways and 3D motion capture systems, which allow multidimensional gait analysis and provide insights into motor dysfunction mechanisms [88-90].

Protocols for gait speed measurement in MCR remain inconsistent, and standardized approaches are lacking. Reported measurement distances vary substantially (2-8 meters for manual methods and 2.4-30 meters for instrumented approaches) [91, 92], and consensus on diagnostic cutoff values remains limited. Alternative functional tests (e.g., five-times-sit-to-stand) are under investigation but require further validation. This variability limits comparability across studies and underscores the importance of developing harmonized protocols.

To improve consistency, we recommend adopting a standardized protocol using a 4-meter walking distance, with 2-meter acceleration and deceleration zones at both ends. Participants should walk at a normal, comfortable pace, and two consecutive trials should be performed, with the average used for analysis to enhance reliability [93].

Gait speed is commonly dichotomized using population-specific thresholds, such as 0.80 m/s. A widely accepted method defines slow gait as a walking speed ≥1 SD below the age- and sex-specific mean, which accounts for demographic and protocol variations across populations [94, 95]. Age-stratified cutoffs include 0.74 m/s for individuals <75 years and 0.60 m/s for those ≥75 years, while sex-specific thresholds are 0.67 m/s for males and 0.51 m/s for females [96, 97]. Considerable heterogeneity persists across studies, and no protocol has yet been universally adopted. A recent systematic review noted that factors such as trial length, averaging versus peak speed, and handling of acceleration/deceleration phases can significantly affect results, emphasizing the need for further standardization before prescriptive thresholds can be recommended [98].

### 6.2 Methods and Standards for Assessing SCD

SCD often precedes MCI and refers to self-perceived decline in memory or other cognitive functions despite normal objective test performance. Several tools exist for SCD assessment, generally categorized as unidimensional or multidimensional. The choice of method directly influences screening accuracy and comparability across MCR studies.

SCD assessment remains methodologically heterogeneous, limiting comparability between studies and complicating standardized diagnosis. Unidimensional assessments usually rely on a single memory-related question, and most studies evaluate only self-reported SCD without input from informants or clinicians. Most studies classify SCD using a single item, and the majority apply no more than four questions. The memory item of the Geriatric Depression Scale (GDS)—“*Do you feel you have more memory problems than most people?*”—is the most frequently applied single-question measure of SCD [99]. However, growing evidence indicates that multi-item assessments covering several cognitive domains provide stronger predictive value for cognitive decline and dementia in older adults with slow gait [100]. Findings from Chinese cohorts further show that cultural and linguistic factors affect the validity of self-reported SCD, highlighting the need for culturally adapted tools [99].

Emerging data link MCR with deficits in attention, memory, and overall cognitive function, emphasizing the importance of multidimensional assessments that incorporate evaluations of executive function and working memory - domains essential for accurate MCR diagnosis and management. For example, one study applied the validated 40-item Cognitive Change Index (CCI-40) to assess memory, executive function, attention, language, and visuospatial abilities using a 5-point Likert scale, where higher scores indicated more severe perceived cognitive difficulties. This study found that SCD assessed using the CCI-40 significantly predicted future dementia risk in older adults with slow gait, whereas the predictive value was not significant in those with normal gait [101]. Nonetheless, most current MCR studies continue to use overly simple SCD assessments, often a single question, which limits accurate identification of high-risk individuals [100].

The Global Subjective Cognitive Decline Scale integrates self-reported data across multiple domains, enhancing the specificity of SCD assessments. Although multidimensional tools improve diagnostic accuracy, their longer administration time limits widespread clinical use. This creates a trade-off between research precision and feasibility, requiring researchers and clinicians to choose assessment tools that balance both.

## 7. MCR and Adverse Health Outcomes

MCR is associated with increased risk of dementia progression, falls, mortality, and functional decline across multiple systems [6]. Early recognition and timely intervention may help improve prognosis and mitigate disability in older populations.

### 7.1 MCR as a Predictor for Dementia Risk

MCR is recognized as an important predementia syndrome. Longitudinal studies consistently show that individuals with MCR have a higher risk of cognitive decline and subsequent dementia.

Evidence on MCR prevalence and its predictive value for dementia demonstrates that a large multi-country study involving 26,802 adults aged 60 and older free from dementia and disability across 17 countries identified MCR as a strong and early risk factor for both cognitive decline and dementia onset. The study reported an adjusted hazard ratio (aHR) of 2.0 (95% CI = 1.7-2.4) for predicting cognitive impairment and an aHR of 1.9 (95% CI = 1.5-2.3) for predicting dementia [5]. These associations remained significant even after excluding individuals with potential cognitive impairment, adjusting for early dementia, and accounting for diagnostic overlap with other predementia syndromes. These findings support the use of MCR as a clinical marker to identify high-risk older adults across populations. Additionally, a nationwide cohort study in Korea involving 1,137,530 individuals aged 66, with a mean follow-up of 7.02 years, found that MCR subtypes characterized by subjective cognitive complaints combined with either Timed Up-and-Go (TUG) impairment or one-leg-standing (OLS) impairment were associated with approximately a two-fold increased risk of developing dementia, compared to individuals without MCR (TUG subtype aHR = 2.03, 95% CI = 1.94-2.13; OLS subtype aHR = 2.05, 95% CI = 1.98-2.12) [102]. This study further showed that MCR provides greater predictive validity for dementia onset than assessments of cognitive or motor impairment alone.

Although MCR is classified as a motoric-based predementia syndrome, evidence indicates that the severity of cognitive impairment, rather than motor dysfunction alone, better predicts progression to dementia. A recent multicenter study involving 610 older adults with MCR from three U.S.-based cohorts followed over a mean of 2.9 years found that poorer logical memory, greater severity of cognitive complaints, and lower Mini-Mental State Examination (MMSE) scores significantly predicted conversion to dementia, while gait velocity did not independently predict progression [103]. In addition to core symptoms, other predictors of MCR progression include depressive symptoms and the presence of the *APOE* ε4 allele. Conversely, higher participation in cognitive activities may reduce the risk of conversion [103, 104]. These associations suggest shared pathological mechanisms underlying cognitive, motor, and mood changes in aging. Further investigations have classified MCR into subtypes based on specific gait parameters, each exhibiting distinct neuropsychological profiles and risk factors. For instance, the velocity-dominant subtype (MCRv) is linked to global cognitive decline, particularly in attention and language domains, and predicts incident impairment in overall cognition. In contrast, the swing-time-variability subtype (MCRswv) is specifically associated with memory deficits and predicts future memory-related cognitive impairment. These divergent cognitive profiles and risk factor associations indicate heterogeneous mechanisms within MCR and may inform more targeted strategies for early detection and intervention [105].

### 7.2 MCR and Fall Risk

MCR, defined by subjective cognitive complaints (SCC) and slow gait, is recognized not only as a prodromal stage of dementia but also as a predictor of falls in older adults. Longitudinal studies show that MCR predicts future fall risk. For example, a Chinese study of 3,748 older adults found that MCR independently increased the risk of falls over the following three years by 66.7% (OR = 1.51, 95% CI = 1.08-2.12) compared to individuals without MCR. In that study, SCC alone also significantly predicted falls (OR = 1.24, 95% CI = 1.01-1.51), whereas slow gait alone did not remain an independent predictor after full adjustment [106]. Additional support comes from a large-scale prospective study involving over 10,000 participants from the ELSA, HRS, and CHARLS cohorts, which confirmed MCR’s association with increased fall risk over a four-year period. Relative risk increases of 60.00%, 50.50%, and 34.10% were reported in the respective cohorts. This multi-cohort analysis confirmed MCR as a consistent predictor of future falls, whereas slow gait alone did not show an independent association across cohorts [107].

The relationship between MCR and falls may be bidirectional; evidence suggests that falls can also precede the development of MCR. A longitudinal study of 3,720 Chinese participants found that experiencing a single fall nearly doubled the risk of developing MCR over time (HR = 2.02, 95% CI = 1.03-3.96) [108]. This bidirectional association indicates a complex interplay between motor and cognitive systems. Individuals with MCR, due to concurrent gait and cognitive impairments, face greater difficulty with environmental adaptation, motor control, and postural balance—factors that heighten their fall risk. Longitudinal studies also report a bidirectional relationship between gait speed and cognition: slower gait may accelerate cognitive decline, while cognitive impairment can further worsen gait [109]. For example, findings from the CHARLS cohort in China show that baseline cognition predicts subsequent gait changes, and vice versa [110]; similar interactive associations have also been reported in U.S. cohorts. These findings support the hypothesis of a reinforcing feedback loop between gait and cognition [103]. Neuroimaging studies support this mechanism, showing that individuals with MCR exhibit neurodegenerative changes such as hippocampal atrophy, weakened frontotemporal network connectivity, and cerebral small vessel disease. These structural abnormalities disrupt prefrontal-basal ganglia circuits, leading to increased stride variability and reduced double support phase, both key indicators of gait instability [69].

This combined impairment of cognition and motor function supports the use of MCR as a biomarker for predicting fall risk in older adults.

### 7.3 MCR and Frailty

Frailty is a clinical syndrome marked by reduced physiological reserve and dysfunction across multiple organ systems [43]. Studies have demonstrated a bidirectional relationship between MCR and frailty [111, 112], in which elevated frailty levels increase the risk of developing MCR, while MCR significantly raises the likelihood of frailty in older adults. In individuals with MCR, slow gait and cognitive decline contribute to muscle weakness and a loss of functional independence. Social frailty often precedes psychological and physical frailty, eventually progressing to cognitive frailty [112]. MCR is regarded as a predictor of frailty and subsequent disability in older adults. In a study of 429 Chinese older adults, MCR was independently associated with a 4.53-fold increased risk of frailty (OR = 5.53, 95% CI = 1.46-20.89). Notably, slow gait alone also increased frailty risk by 2.4 times (OR = 3.40, 95% CI = 1.40-8.23) [113]. MCR’s predictive role extends beyond frailty to functional decline. A 7-year longitudinal study found that participants with MCR at baseline had a 58% higher risk of developing ADL disability (OR = 1.58, 95% CI = 1.19-2.09) and a 46% higher risk of Instrumental Activities of Daily Living (IADL) disability (OR = 1.46, 95% CI = 1.13-1.88) compared to those without MCR. This study also showed that early detection of MCR is valuable, as the memory-impairment subtype (MCR-MI) carried a higher risk of future IADL disability (OR = 2.14, 95% CI = 1.18-3.88) [114].

### 7.4 MCR and Dysfunction of Other Organ Systems

MCR is influenced by comorbid health conditions, particularly cardiovascular diseases and multimorbidity. These conditions can contribute to both the onset and progression of MCR symptoms. Research has shown a significant association between cardiometabolic multimorbidity (CMM)—defined as the presence of two or more cardiometabolic diseases such as diabetes, heart disease, or stroke—and an increased risk of developing MCR. A longitudinal study of 4,744 participants aged 65 and older found that CMM raised the risk of MCR by 41% (HR = 1.41, 95% CI = 1.13-1.75). This association followed a dose-dependent pattern: each additional cardiometabolic condition further elevated the risk of developing MCR (HR = 1.33, 95% CI = 1.20-1.48) [45].

Beyond cardiometabolic disorders, general multi-morbidity, defined as the co-occurrence of multiple chronic conditions, is also highly prevalent among older adults who develop MCR. A study involving 4,923 Chinese participants found that multimorbidity significantly increased the risk of MCR (HR = 1.33, 95% CI = 1.06-1.68). Additionally, the number of comorbid conditions was positively correlated with MCR risk (HR = 1.10, 95% CI = 1.02-1.19). Notably, individuals with a cardiovascular multimorbidity pattern were 1.57 times more likely to develop MCR compared to those with relatively healthy profiles (HR = 1.57, 95% CI = 1.16-2.13) [73]. These findings suggest that comorbidities may accelerate MCR development through mechanisms such as neurodegeneration and systemic inflammation, contributing to poorer prognosis.

### 7.5 MCR and the Risk of Mortality

Multiple studies have shown an association between MCR and increased all-cause mortality. A meta-analysis involving 1,224,569 participants reported that individuals with MCR had a 66% higher risk of death (adjusted Relative Risk [RR] = 1.66, 95% CI = 1.32-2.10) [6].

Individual cohort studies support this finding. For example, one study including 11,867 non-demented individuals over the age of 65 from three established cohorts in the United States and Europe found that MCR was significantly associated with increased overall mortality (adjusted Hazard Ratio [aHR] = 1.69, 95% CI = 1.46-1.96) and with 2-year mortality (adjusted Odds Ratio [aOR] = 1.89, 95% CI = 1.50-2.38) [115, 116]. The predictive value of MCR for mortality appears independent of baseline gait speed and cognitive scores [116], supporting its role as a composite biomarker that reflects risk dimensions beyond traditional measures [103]. Current evidence is observational, and although associations are strong, causality cannot be confirmed without further mechanistic or interventional studies.

## 8. Therapeutic Strategies for MCR: Pharmacological and Non-pharmacological Approaches

**Figure 3.**
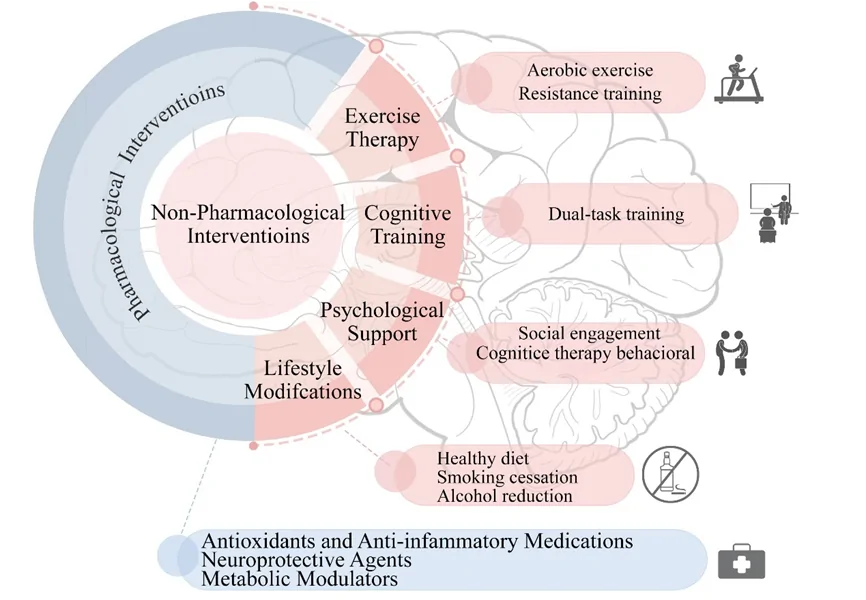
**Interventions and Preventive Measures for MCR**. Intervention strategies, including non-pharmacological approaches such as exercise therapy, cognitive training, and lifestyle modifications, as well as pharmacological measures, are summarized. Abbreviations: MCR, Motoric Cognitive Risk Syndrome.

Interventions for MCR include pharmacological options (e.g., antioxidants, neuroprotective agents, metabolic modulators) and non-pharmacological strategies (e.g., exercise, cognitive training, lifestyle modifications), aimed at improving cognitive, motor, and functional outcomes (Table 2). An overview of these interventions is provided in Figure 3. While pharmacological treatments may address specific symptoms, evidence to date more consistently supports multimodal non-pharmacological interventions. For example, aerobic exercise can improve cerebral oxygenation, dual-task training may enhance cognitive—motor integration, and adherence to a Mediterranean diet has been linked to reduced systemic inflammation [38].

**Table 2:** Interventions and Preventive Measures for MCR.

Intervention Type	Specific Measures	Research Findings
Pharmacological	Antioxidants and Anti-inflammatory Medications	Vitamin E improves cognition in AD and delays functional decline in some RCTs, but evidence in MCR populations is lacking. Well-designed RCTs are needed to test efficacy and safety specifically in MCR [117]. NSAIDs and biologics may reduce inflammation, yet require long-term safety evaluation before clinical adoption [118].
	Neuroprotective Agents	Memantine and cholinesterase inhibitors are effective in AD but lack direct interventional evidence in MCR. Randomized or longitudinal intervention trials are required to establish their role in MCR [119].
	Metabolic Modulators	Metformin and PPAR-γ agonists may benefit individuals with MCR with metabolic comorbidities [120]. Evidence is mostly extrapolated from non-MCR populations; targeted RCTs in MCR are needed. Nutritional supplements (omega-3, B vitamins, vitamin D) show neuroprotective effects but require validation through controlled trials in MCR.
Non-Pharmacological	Exercise Therapies	Aerobic, resistance, balance, and multimodal exercise improve gait, cognition, and reduce fall risk [121]. The ENGAGE-B study showed feasibility of a 24-week multimodal program [52]. Larger, long-term community-based RCTs are required to confirm effectiveness in MCR.
	Cognitive Training	Computerized and dual-task training improve cognition, gait stability, and reduce frailty [122]. Mechanistic neuroimaging evidence suggests enhanced cortical activity. Future trials should examine durability of effects and scalability in MCR populations.
	Psychological Supports	Depression is an independent risk factor for MCR (CHARLS study). SSRIs, CBT, and counseling improve mood and may preserve cognition [123]. More evidence from controlled interventional studies is needed to establish long-term cognitive benefits in MCR.
	Lifestyle Modifications	Healthy diet, smoking cessation, and reduced alcohol intake lower cardiovascular risk and indirectly reduced MCR. Moderate naps (30-89 min) are associated with lower MCR prevalence, but causal relationships need validation in prospective cohorts.

Abbreviations: AD, Alzheimer's Disease; RCT, Randomized Controlled Trial; MCR, Motoric Cognitive Risk Syndrome; PPAR-γ, Peroxisome Proliferator-Activated Receptor Gamma; CHARLS, China Health and Retirement Longitudinal Study; SSRIs, Selective Serotonin Reuptake Inhibitors; CBT, Cognitive-Behavioral Therapy.

### 8.1 Pharmacological Interventions

#### 8.1.1 Antioxidants and Anti-inflammatory Medications

Vitamin E exhibits multifaceted effects on cognitive health. Clinical trials have demonstrated that it improves cognitive function in patients with moderate-to-severe AD [117], and most epidemiological studies support an association between higher vitamin E levels and both cognitive enhancement and reduced AD risk. Mechanistic studies highlight oxidative stress as a contributing factor to cognitive decline [124], and specific members of the vitamin E family, such as tocotrienols (T3) [125], have shown potential to counteract these processes. Although several randomized controlled trials (RCTs) report that vitamin E supplementation can safely delay functional decline in AD patients, other studies have failed to replicate these benefits in relation to cognitive deterioration [126]. Moreover, no interventional studies have yet evaluated vitamin E’s effects specifically in populations with MCR syndrome. However, given its favorable outcomes in AD-related RCTs, along with its strong safety profile and cost-effectiveness, vitamin E remains a potential candidate for neuro-nutritional intervention. Further standardized clinical research is needed to clarify its role in promoting healthy brain aging, slowing AD progression, and determining its efficacy across various AD stages and in individuals with MCR.

Chronic inflammation is considered an important pathological mechanism of MCR, with evidence showing that elevated CRP and IL-6 levels are closely associated with increased MCR risk [127]. Although observational studies have suggested that long-term NSAID use is linked to a reduced risk of cognitive decline, multiple randomized controlled trials (RCTs) have failed to confirm their preventive or therapeutic efficacy, and NSAIDs are also associated with an increased risk of gastrointestinal bleeding and cardiovascular adverse events [118, 128]. Thus, although anti-inflammatory drugs may offer theoretical benefits, their use in MCR requires careful evaluation of efficacy and safety. Future targeted RCTs and mechanistic studies are needed to evaluate their risk-benefit profile.

#### 8.1.2 Neuroprotective Agents: Cognitive Improvement Drugs

Neuroprotective agents, such as memantine and cholinesterase inhibitors (e.g., donepezil), improve cognitive function by preserving neural transmission. Although commonly used in AD [119, 129], these agents hold theoretical potential to slow cognitive decline in individuals with MCR. However, direct interventional evidence in MCR populations remains limited and warrants further investigation.

#### 8.1.3 Metabolic Modulators

Metabolic modulators, including metformin and Peroxisome Proliferator-Activated Receptor Gamma (PPAR-γ) agonists, enhance insulin sensitivity and metabolic function [120]. These interventions show promises for slowing cognitive and motor decline in individuals with MCR with metabolic comorbidities, but more direct evidence in this population is needed.

Nutritional supplements offer additional benefits: omega-3 fatty acids reduce neuroinflammation [62], B vitamins lower homocysteine levels to support brain function [69], and vitamin D may improve cognition by promoting neuroprotection, reducing inflammation, supporting nerve growth, and maintaining calcium balance.

While antioxidants, anti-inflammatory agents, neuroprotective drugs, and metabolic modulators show potential as interventions, rigorous studies are necessary to confirm their efficacy and safety specifically in MCR populations. Such evidence will be essential for developing effective clinical management strategies.

### 8.2 Non-Pharmacological Interventions

#### 8.2.1 Exercise Therapy

Physical exercise benefits brain health and cognitive function, primarily through the upregulation of brain-derived neurotrophic factor (BDNF), improvements in cerebral blood flow, and structural changes in key brain regions, including the hippocampus, white matter, gray matter, and cortical thickness. Exercise training has been shown to reduce anxiety and depression and improve multiple cognitive domains, including memory, attention, executive function, processing speed, language, motor coordination, and visuospatial skills [121]. Literature consistently indicates that high- and moderate-intensity aerobic exercise yields the most significant cognitive benefits. Emerging evidence also supports the positive effects of resistance training, dual-task training, and mind-body practices [43].

Multimodal physical activity is a key component of MCR intervention and includes aerobic exercise (e.g., brisk walking, swimming), resistance training, flexibility exercises, and balance training. Current guidelines recommend that older adults engage in 3-5 sessions of moderate-intensity exercise per week, each lasting 30-60 minutes. These activities consistently improve gait and cognitive function, thereby reducing the risk of MCR [43]. Research has shown that moderate-intensity walking, resistance training, flexibility exercises, and combined aerobic and resistance programs enhance gait, cognitive performance, neurological health, motor function, and the ability to perform activities of daily living. Traditional exercises such as Tai Chi, Baduanjin, and dancing have also been associated with improvements in cognitive and motor function in individuals with MCR. Beyond improving physical strength, exercise training reduces fall risk by enhancing balance and body control. Notably, exercise prescriptions for individuals with MCR should be individualized according to their functional status, cognitive characteristics, and comorbid conditions. For example, those with relatively preserved function may benefit more from moderate-intensity aerobic exercise and dual-task training, whereas individuals with MCR who present with gait instability or a history of falls may require low-intensity, balance-focused supervised programs to ensure safety. The ENGAGE-B study demonstrated the initial feasibility of delivering a 24-week multimodal physical activity (PA) intervention for older adults with MCR in community settings, laying the foundation for future large-scale, effective lifestyle intervention trials targeting MCR [52]. In addition to physical activity, good nutrition, such as diets rich in vitamin D3, calcium, or blueberries, is associated with improved gait, better cognitive performance, and a lower risk of dementia [16]. Overall, these findings highlight the importance of integrating exercise and nutritional interventions in the management of MCR.

#### 8.2.2 Cognitive Training

Cognitive training aims to improve cognitive function in older adults through targeted interventions, which may help prevent MCR and reduce fall risk [130]. One primary approach is computerized cognitive training (CCT), which enhances cognitive performance through repetitive exercises targeting memory, attention, and executive function. For instance, one study reported that participants using the Brain Age computer training system showed significant improvements in overall cognitive, executive, and motor functions [122].

In addition, dual-task training—which combines cognitive challenges with physical activity—can improve mobility in older adults and reduce fall risk. These programs typically involve 2-3 sessions per week, each lasting about 30 minutes, over an 8-12 week period. The training progresses from simple tasks (e.g., counting while walking) to more complex exercises involving calculations or memory challenges. Notably, the HAPPY exercise program, conducted in community settings, devotes up to two-thirds of its 60-minute sessions to dual-task activities. During these sessions, participants simultaneously march, clap, and solve arithmetic problems (e.g., addition or subtraction), thereby engaging multiple brain regions. This training significantly increases activity in Broca’s area, its right-hemisphere counterpart, and across the fronto-temporo-parietal cortex [7].

Dual-task training has been shown to reduce frailty, improve attention and cognitive performance, and enhance gait. These benefits arise from improved biomechanics, strengthened neuromuscular control, promoted neuroplasticity, and brain remodeling. Together, these adaptations may help reduce fall risk in individuals with MCR [43].

#### 8.2.3 Psychological Support

MCR impairs quality of life, independence, and social engagement in older adults. Social interaction has emerged as a modifiable factor that supports both cognitive and psychological health. Providing psychological support is important for maintaining emotional well-being in individuals with MCR. According to the CHARLS study, depression is an independent risk factor for MCR among community-dwelling older adults in China. Both antidepressant treatment and psychological counseling can effectively alleviate depressive symptoms [131].

Studies further suggest that antidepressant medications, particularly selective serotonin reuptake inhibitors (SSRIs), not only relieve emotional distress but also help preserve cognitive function. Additionally, psychological interventions such as cognitive-behavioral therapy (CBT) reduce maladaptive thinking patterns, enhance self-efficacy, and promote social interaction [130].

#### 8.2.4 Lifestyle Modifications

Lifestyle changes, including a healthy diet, smoking cessation, and reduced alcohol consumption, can indirectly slow MCR progression by lowering cardiovascular risk. Evidence from the CHARLS study also indicates that moderate napping (30-89 minutes) may reduce MCR prevalence, especially in older adults who sleep ≤8 hours per night [76].

## 9. Conclusion

Screening and early intervention for MCR are clinically valuable, offering an opportunity to delay cognitive decline and improve quality of life. Large-scale cohort studies play a key role in clarifying the epidemiological characteristics and progression of MCR across diverse populations. Multicenter long-term follow-up studies are also needed to identify group-level differences in cognitive and motor decline, supporting the development of more effective diagnostic and treatment strategies.

Current research efforts focus primarily on two key areas. First, there is increasing interest in identifying early MCR biomarkers using advanced multi-omics technologies, including proteomics and metabolomics. Second, researchers are working to develop and validate novel assessment tools. For example, machine learning approaches have shown strong potential in predicting cognitive decline in individuals with MCR by integrating multi-omics data (e.g., plasma β-amyloid/tau levels) with functional assessments (e.g., dual-task TUG parameters) [132]. Although these models show promising predictive performance—one study reported an AUC of 0.82—their generalizability is limited by small sample sizes. To address this limitation, future research must prioritize large-scale validation studies. These efforts will be important for developing a comprehensive MCR early-warning system that integrates multi-omics data with functional assessments.

Emerging technologies, including virtual reality (VR) and AI-assisted cognitive training, hold promise for future MCR management. However, the clinical application of AI still faces significant challenges, including data bias, limited model interpretability, and the need for rigorous external validation. Establishing effective human-machine collaboration frameworks is also critical. Ethical considerations should guide implementation, ensuring that clinicians and researchers avoid premature labeling, allocate medical resources judiciously, and clearly differentiate between “risk status” and “disease diagnosis.” To ensure transparency and patient autonomy, the development of standardized protocols is recommended, including dynamic informed consent processes monitored by institutional ethics committees.

Through multidisciplinary collaboration and the integration of advanced technologies, more precise systems for early MCR detection and intervention are expected to emerge. These advances may yield novel strategies to delay cognitive decline and enhance quality of life in at-risk populations.
